# Latent Profile Analysis of AI Literacy and Trust in Mathematics Teachers and Their Relations with AI Dependency and 21st-Century Skills

**DOI:** 10.3390/bs14111008

**Published:** 2024-10-30

**Authors:** Tommy Tanu Wijaya, Qingchun Yu, Yiming Cao, Yahan He, Frederick K. S. Leung

**Affiliations:** 1 School of mathematical Sciences, Beijing Normal University, Beijing 100088, China; yuqc@bnu.edu.cn (Q.Y.); caoym@bnu.edu.cn (Y.C.); 2 National Research Institute for Mathematics Teaching Materials, Beijing 100190, China; 3 School of Mathematical Sciences, Capital Normal University, Beijing 100048, China; b454@cnu.edu.cn; 4 College of Education for the Future, Beijing Normal University, Zhuhai 519087, China

**Keywords:** ChatGPT, AI tools, latent profile analysis, dependency, 21st-century skills

## Abstract

Artificial Intelligence (AI) technology, particularly generative AI, has positively impacted education by enhancing mathematics instruction with personalized learning experiences and improved data analysis. Nonetheless, variations in AI literacy, trust in AI, and dependency on these technologies among mathematics teachers can significantly influence their development of 21st-century skills such as self-confidence, problem-solving, critical thinking, creative thinking, and collaboration. This study aims to identify distinct profiles of AI literacy, trust, and dependency among mathematics teachers and examines how these profiles correlate with variations in the aforementioned skills. Using a cross-sectional research design, the study collected data from 489 mathematics teachers in China. A robust three-step latent profile analysis method was utilized to analyze the data. The research revealed five distinct profiles of AI literacy and trust among the teachers: (1) Basic AI Engagement; (2) Developing AI Literacy, Skeptical of AI; (3) Balanced AI Competence; (4) Advanced AI Integration; and (5) AI Expertise and Confidence. The study found that an increase in AI literacy and trust directly correlates with an increase in AI dependency and a decrease in skills such as self-confidence, problem-solving, critical thinking, creative thinking, and collaboration. The findings underscore the need for careful integration of AI technologies in educational settings. Excessive reliance on AI can lead to detrimental dependencies, which may hinder the development of essential 21st-century skills. The study contributes to the existing literature by providing empirical evidence on the impact of AI literacy and trust on the professional development of mathematics teachers. It also offers practical implications for educational policymakers and institutions to consider balanced approaches to AI integration, ensuring that AI enhances rather than replaces the critical thinking and problem-solving capacities of educators.

## 1. Introduction

In recent years, AI technology has undergone substantial development and has been increasingly implemented in the education sector [1,2]. This technology has proven to offer numerous new opportunities and has a positive impact on learning activities [3]. AI enables both teachers and students to innovate in their teaching and learning practices, fostering a more interactive and dynamic educational environment [4,5]. The integration of AI tools in Wolfram Alpha, Julius, Maple calculator, and Microsoft math solver, for example, facilitates personalized learning experiences, enhances engagement through interactive content, and streamlines administrative tasks, thereby allowing educators to focus more on pedagogy. Studies have shown that the use of such generative AI tools in teaching can significantly enhance teachers’ pedagogical skills by providing them with real-time feedback and actionable insights, leading to more informed instructional decisions [2,6].

The application of AI technology has been particularly notable in the field of mathematics education. Here, AI assists teachers in various aspects such as generating mathematics problems, crafting detailed lesson plans, and providing real-time feedback to students [7]. The effectiveness of these AI-driven tools, however, is significantly influenced by the level of AI literacy among teachers, which determines their ability to implement these technologies effectively [8,9,10]. Moreover, the trust that teachers place in these tools may impact their willingness to rely on AI for educational tasks [11,12], thereby influencing the overall educational experience, which in turn influences the overall educational experience. Understanding the interplay between AI literacy and AI trust is crucial for maximizing the benefits of AI in education while addressing potential challenges such as increased AI dependency. AI dependency is described as the extent to which teachers rely on AI technologies for teaching and administrative tasks. This variable is influenced by both AI literacy and trust, reflecting the level of integration and reliance on AI in daily educational practices. This dependency can lead to a loss of decision-making abilities and reduced initiative among students and teachers, often perceived as increased laziness [13,14]. As AI assumes more routine tasks, educators and learners may become overly reliant on technology, undermining their capacity to independently solve problems and make informed decisions. Additionally, the widespread adoption of AI requires stringent measures to ensure data privacy and accuracy of content and safeguard against potential biases, thus maintaining the integrity of educational settings.

While there is abundant literature on AI literacy [12,15,16], previous research has primarily focused on the effects of AI literacy on the use of AI technology. This includes studies on how AI literacy influences behavioral intentions to use technology, the impact of AI literacy on work performance, and the effects of AI literacy on AI learning. AI literacy is defined as the knowledge and skills related to understanding, evaluating, and utilizing AI technologies. This encompasses teachers’ ability to integrate AI tools into educational settings effectively and ethically. However, there has been no research that analyzes the profiles of AI literacy and combines them with AI trust to explore their impact on AI dependency and 21st-century skills [15].

This oversight is significant because while the integration of AI into educational settings is often heralded for its potential to enhance learning and efficiency, it also raises substantial concerns about its long-term effects on essential educational outcomes. Critically, the literature lacks a nuanced discussion of the potential negative implications of increasing AI dependency, such as whether reliance on AI tools could diminish teachers’ abilities in problem-solving and critical thinking. This is a crucial area of debate, as some scholars argue that AI can supplement these skills by offloading routine tasks and allowing teachers to focus on more complex pedagogical strategies, while others contend that it may lead to a degradation of these core competencies due to reduced engagement in cognitive processes.

This study aims to fill the research gap by investigating the profiles of AI literacy and AI trust of mathematics teachers, in line with the Technology Acceptance Model [17], which posits that AI literacy and AI trust are multifaceted constructs that influence an individual’s use of technology, in this context, AI technology. Furthermore, it explores the differences among profiles of AI literacy and AI trust of mathematics teachers with respect to AI dependency and 21st-century skills. This study is the first to use the latent profile analysis (LPA) approach [18] to analyze the multidimensionality and complexity of AI literacy and AI trust of mathematics teachers, while also examining differences in AI dependency and 21st-century skills among the profiles. The latent profiles focus on subgroups among the participants, demonstrating distinct patterns across different dimensions of AI literacy and AI trust. This research may contribute to the field of AI education by providing new insights into how the AI literacy and AI trust of mathematics teachers may have strong connections to AI dependency and 21st-century skills. Moreover, the findings on differences in levels of AI literacy and AI trust could assist curriculum developers or schools in providing targeted interventions for different mathematics teachers when using AI technology so that they can enhance their pedagogical strategies and effectively integrate these technologies into their teaching practices.

The two research questions for this study are as follows:

1. What are the latent profiles of AI literacy and AI trust among mathematics teachers in China?
2. Are there significant differences in AI dependency and 21st-century skills across different profiles of AI literacy and AI trust?

The remainder of this paper is organized as follows: The second section provides a comprehensive literature review, which delves into previous studies on AI literacy, trust in AI, and the utilization of AI in educational settings, as well as the theoretical frameworks supporting these concepts. The third section describes the methodology employed in this study, including the use of LPA to identify distinct profiles of AI literacy and trust among mathematics teachers. The fourth section presents the results of the analysis, exploring the relationships between these profiles, AI dependency, and the development of 21st-century skills. The fifth section discusses the implications of these findings for both practice and policy and suggests directions for future research. Finally, the paper concludes with a summary of the key findings and their significance for the field of AI in education

## 2. Literature Review

### 2.1. The Role of AI in Mathematics Education

Mathematics involves the study of concepts such as numbers, quantities, space, and structures through symbolic language [19]. Mathematical education is considered a complex and challenging task aimed at developing learners’ problem-solving abilities [20,21]. With the advancement of big data and AI technologies, the integration of mathematics education and AI is increasingly evident, presenting new challenges and opportunities [22,23]. This trend places higher demands on the AI literacy, AI trust, and 21st-century skills of mathematics teachers. To optimize the teaching process and enhance student learning experiences, mathematics teachers must possess a high level of literacy to fully comprehend and apply AI technologies. Additionally, trust in AI (AI Trust) is crucial [12]. Teachers need to believe in these technologies to ensure their appropriate and effective use in education. Furthermore, 21st-century skills such as critical thinking, creativity, collaboration, and technological literacy have become particularly important [24,25]. Twenty-first-century skills are defined as the skills that students need to develop to succeed in the contemporary world, such as critical thinking, creativity, collaboration, and communication. Teachers must not only possess these skills but also effectively impart them to students, fostering innovative talents to meet future societal needs. Thus, mathematics teachers in this new era face the pressing need to continuously enhance their professional competencies and adapt to technological changes to address the diversity and complexity of future education.

AI involves the simulation of human intelligence processes by machines, particularly computer systems [13,26]. Specific applications of AI within higher education institutions include expert systems [27], natural language processing [8], speech recognition, and machine vision [28]. These technologies are increasingly integrated into university and college campuses, enhancing both administrative efficiency and academic engagement. The rapid development of AI and other disruptive technologies is significantly impacting human society, including the field of education [29,30]. Generative AI, exemplified by ChatGPT [14], has a wide range of applications in education. Teachers are increasingly exploring the potential of generative AI in classroom instruction, including human–machine collaborative creation, dynamically providing instructional frameworks, and offering personalized learning support and tutoring [31]. Moreover, AI learning platforms, educational games, chatbots, virtual tutors, and organizational tools are becoming more prevalent every day [2,32,33,34]. Integrating AI technologies into educational settings allows AI to serve as an intelligent tutor, tool, or student, as well as a facilitator for policy-making. For instance, previous studies have used AI technologies to simulate teachers’ behaviors in diagnosing students’ learning problems and providing personalized learning content, pathways, and guidance for individual students [35,36]. A recent review of technology-enhanced adaptive/personalized learning highlighted that advancements in AI have gradually achieved a critical goal of technology-enhanced learning: providing personalized or adaptive learning environments to improve student performance [37]. One study reported that situational personalization in intelligent tutoring systems (ITSs) could enhance learners’ situational interest and performance in mathematical tasks [32]. Another example involves developing student models using AI technologies, such as unsupervised machine learning methods, to predict individual students’ engagement or status in mathematics courses [16,38].

In the realm of education, notable examples include the official launch of ChatGPT Edu by OpenAI on 31 May 2024 [39], specifically designed for Higher Education Institutions to significantly enhance the efficiency and quality of learning and teaching processes [40]. Another example is the development of the foundational model EduChat by East China Normal University [41], which provides mathematics teaching and learning services, with its code, data, and parameters shared as open-source resources. In the field of mathematics education, MathGPT [42], developed by the TAL Education Group in 2021, focuses on addressing math-related problems and delivering lectures for users worldwide. The use of visualization software to obtain graphics and animations of mathematical representations can enhance students’ imagination and problem-solving skills [43]. Integrating AI technologies into educational settings enables AI-based learning systems to function as intelligent tutors, mathematical tools, or student aids and facilitate policy-making. The use of AI tools can support skills and strategies highly relevant to mathematical content areas, such as solving real-world mathematical problems or visualizing complex relationships [44,45]. These tools could be beneficial for supporting mathematics learning through interactive and scaffolded activities. Furthermore, leveraging generative AI could support model-based learning, as students could gain a more detailed understanding of mathematical concepts by observing the direct consequences of their changes. This approach could help students overcome cognitive limitations arising from various misconceptions, a benefit attributed to the adaptability and responsiveness of generative AI.

### 2.2. Positive Effect of AI Technology in Mathematics Education

When mathematics education encounters the era of AI, profound changes are bound to occur. In this new context, mathematics teaching will become more intelligent, personalized, and efficient, providing students with an entirely new learning experience [46]. AI will transform the methods of mathematics instruction. Traditional methods are often unidirectional, with teachers delivering knowledge in the classroom and students passively receiving it [47]. However, with the aid of AI, mathematics instruction will become more personalized and diverse in its interactive forms [48]. Intelligent teaching systems can tailor learning plans to students’ individual learning conditions and ability levels, providing personalized mathematics learning resources and practice problems [49,50]. As a result, students who struggle with learning can follow customized learning paths and more efficiently master mathematical knowledge [51]. Supporting this shift, the recent UNESCO AI competence framework for teachers outlines essential skills and knowledge educators need to effectively integrate AI into their teaching practices, further driving the evolution of educational methodologies [52].

In addition, AI will enrich mathematics learning resources. In traditional mathematics education, students typically rely on textbooks and teachers’ explanations for learning. In the AI era, students can easily access a wealth of mathematics learning resources through intelligent search and personalized recommendation systems [31,53]. Whether it is the derivation of mathematical formulas, problem-solving approaches, or practical applications of mathematics, AI can help find relevant materials and information [54,55]. This will greatly broaden students’ learning horizons, aiding them in better understanding and applying mathematical knowledge.

Moreover, AI may enhance the mathematics learning experience [56,57]. Mathematics is often considered an abstract and tedious subject, causing many students to feel intimidated and bored. However, with the help of AI, mathematics learning will become more engaging and vivid [58]. Through virtual reality and augmented reality technologies, students can conduct mathematical experiments and explorations in virtual environments [59], intuitively experiencing mathematical concepts and principles. Simultaneously, intelligent assessment systems can provide immediate feedback and suggestions, helping students promptly identify and correct errors, thus improving learning outcomes [60]. This interactive and real-time-feedback learning experience will stimulate students’ interest [61] and motivation [62], making them more proactive in their mathematics studies.

Additionally, AI will promote equity and accessibility in mathematics education. In traditional teaching models, factors such as geography and economics limit many students’ access to quality mathematics education resources. AI technologies can bridge these gaps by providing scalable and personalized learning experiences that are not bound by location or the availability of expert teachers. For example, AI-powered platforms can deliver tailored tutoring and access to a wealth of digital resources, ensuring that all students, regardless of their socioeconomic status or geographical location, have the opportunity to excel in mathematics. However, in the AI era, these limitations will be overcome. As long as there is an internet connection, students can access intelligent teaching systems anytime, anywhere, obtaining the same learning opportunities and resources as those in metropolitan areas [63]. This will help bridge the educational gap and achieve educational equity [64]. In conclusion, the advent of AI in mathematics education will bring about many profound changes. AI will transform teaching methods, enrich learning resources, enhance the learning experience, and promote equity and accessibility in mathematics education. However, it is important to recognize that AI is merely a tool; true mathematics education still requires teachers’ guidance and students’ effort. Only by appropriately integrating AI technologies with traditional educational methods and teaching philosophies can the development and progress of mathematics education be effectively advanced.

### 2.3. AI Literacy Among Teachers

The rapid development of AI is reshaping traditional educational paradigms, prompting ongoing reflection on how to address the opportunities and challenges presented by AI. Cultivating AI literacy among students is inseparable from teachers; thus, enhancing AI literacy among teachers has become an increasingly important topic in the field of education [16,65]. In March 2019, UNESCO published the report “Artificial Intelligence in Education: Challenges and Opportunities for Sustainable Development”, which clearly stated that AI technology would be widely applied in future classrooms, necessitating AI literacy among teachers [66]. This literacy needs to be developed through both pre-service education and in-service training.

The term AI literacy was introduced by scholars as early as the 1970s, initially emphasizing the literacy structure of AI professionals, but it did not gain widespread attention [67]. It was not until the 2020s, with the increasing application of AI in education, that AI literacy re-entered public discourse and sparked extensive research within the educational community [2,16]. The term AI literacy appears variably in academic literature and policy documents as AI literacy, AI competence, AI skills, and AI capability [9,10]. For the purposes of this study, these terms will be collectively referred to as AI literacy. Most studies construct AI literacy frameworks from three dimensions. For example, Kong et al. [68] define AI literacy as comprising “AI concepts, AI applications, and AI ethics”, where “AI concepts” refer to basic AI knowledge and origins, “AI applications” refer to the real-world application of AI technologies, and “AI ethics” refer to the ethical challenges and safety issues encountered in AI applications. Similarly, Kim et al. [69] construct an AI literacy model from the dimensions of “AI knowledge, AI skills, and AI attitudes”, where “AI knowledge” refers to core AI concepts, “AI skills” refer to computational thinking abilities in AI applications, and “AI attitudes” refer to the ability to critically consider the social impact of AI technologies and appropriately view the relationship between humans and AI.

The definition of AI literacy varies across existing studies. Summarizing current research, there are two primary perspectives on AI literacy. One perspective views AI literacy as a basic ability for teachers to adapt to the AI era, considering teachers as ordinary members of society who should possess general AI knowledge, skills, and awareness [70]. AI literacy includes the ability to critically evaluate AI, communicate and collaborate effectively with AI, and use AI as a learning tool in various contexts such as online, at home, and in the workplace, which is a widely accepted definition of teacher AI literacy [71]. Similarly, UNESCO has developed a competence framework for students that outlines the AI skills and knowledge students need to navigate and succeed in a technology-driven world. This framework complements the teacher-focused guidelines by emphasizing student capacities in understanding, utilizing, and ethically interacting with AI technologies [72].

Another perspective considers TAIL as the basic instructional capability for teachers to adapt to the AI era, which includes the knowledge and skills necessary for integrating AI into daily teaching activities. Zhao et al. [73] argue that the AI literacy of teachers should encompass knowing and understanding AI, applying AI, evaluating AI applications, and understanding AI ethics. Biagini et al. suggest that AI literacy includes four dimensions: Knowledge-related, Operational, Critical, and Ethical. In summary, both perspectives incorporate elements of AI teaching knowledge, AI instructional skills, and attitudes towards AI in education [74]. Although the research focuses differ, both agree that AI literacy is essential for teachers to thrive in an intelligent society and is crucial for their professional development.

### 2.4. Dependency on AI Tools

AI-powered learning technologies, while praised for enhancing efficiency and personalization in education, also raise concerns about increasing technological dependence [75]. Although these tools can automate tasks like providing personalized reminders and real-time feedback, there is growing evidence that such automation may reduce students’ and teachers’ ability to operate independently. For instance, a study involving 1625 students across 10 courses revealed that while AI assistance in peer review initially enhances submission quality, it also fosters a reliance that persists even when AI support is withdrawn, with students showing reduced initiative and poorer self-regulation [76].

This dependency extends to teachers, where AI’s capability to handle repetitive tasks might allow more focus on professional innovation, yet paradoxically, it could also diminish teachers’ innovative capacities by making them overly reliant on technology for educational interactions [77]. Education aims to cultivate “innovative talents”, requiring teachers to lead and guide students’ innovative development through their professional growth [78]. Teachers need to enhance their innovative abilities and learn to use AI technology effectively in teaching activities to achieve professional innovation, thereby fostering educational innovation [79]. However, excessive reliance on AI may weaken teachers’ innovative capabilities [80]. If AI guides all learning processes, students may lose the ability to think independently and solve problems. They might become overly dependent on machines, neglecting the importance of self-exploration and innovation [81]. Moreover, excessive dependence on AI can cause teachers to focus solely on specific issues and fields, overlooking the systematic and holistic nature of knowledge. This fragmented approach to knowledge acquisition hinders the establishment of a comprehensive knowledge system and the development of interdisciplinary thinking among teachers [82].

While AI enhances teaching and learning by automating assessments and reducing the evaluative workload for teachers, it also presents challenges. Over-reliance on AI may erode teachers’ evaluative capabilities and problem-solving skills, leading them to depend too heavily on AI for instructional decisions. This dependency could diminish their professional judgment, with AI’s lack of consciousness potentially impacting the motivational aspects of educational assessments. Furthermore, although AI mimics human intelligence, it cannot replicate human emotions and values or foster genuine interactions, which are crucial for nurturing students’ moral and creative development. The use of AI, such as ChatGPT, might also introduce biases and ethical issues, affecting the quality of interaction and collaboration in the classroom. These concerns suggest that while AI can support educational processes, it should not replace the essential human elements of teaching. An initial hypothesis of our study is that higher AI literacy among teachers may reduce AI dependency. By equipping teachers with the necessary knowledge and skills, AI literacy enables them to integrate AI tools more judiciously, promoting a balanced use of technology in educational settings.

### 2.5. The Importance of 21st-Century Skills

In the context of 21st-century globalization and information technology, education systems must develop teachers with a range of core skills to meet the demands of high-quality talent in modern society [15]. In recent years, scholars have extensively studied and discussed the definition and elements of 21st-century skills for teachers. The framework proposed by the Partnership for 21st Century Learning (P21), a national organization founded in the United States in 2002, is the most widely accepted in this area. P21 collaborates with educators, business leaders, and government officials to design and implement a state-of-the-art framework for 21st-century learning. The P21 framework emphasizes the importance of integrating core academic subjects with essential life and career skills, and it comprises four core areas: Core Subjects and 21st Century Themes, Learning and Innovation Skills, Information, Media, and Technology Skills, and Life and Career Skills. This framework aims to prepare students for the dynamic and interconnected world of the 21st century by ensuring they acquire the necessary skills and knowledge [83]. These shared elements reflect the demands of the AI era for teachers’ 21st-century skills, specifically including self-confidence, problem-solving, critical thinking, creative thinking, and collaboration [84,85]. This study focuses on these five core elements.

Teachers’ self-confidence is a crucial component of 21st-century skills, playing an essential role in the educational process [86]. Current research indicates that confident teachers can more effectively engage students and enhance teaching outcomes [87]. Teachers with high self-confidence maintain a positive attitude when facing teaching challenges and adopt innovative teaching methods, thereby improving student learning outcomes and engagement [88]. Self-confidence also influences teacher–student relationships, promoting students’ psychological well-being and academic performance [89]. Confident teachers can establish positive teacher–student relationships, creating a conducive learning environment that supports students’ holistic development. This study focused on designing an effective professional development program (PDP) to enhance teachers’ confidence and willingness to adopt AI educational technologies.

Teachers’ problem-solving ability is particularly important in managing complex teaching environments, and academic research on this ability has been ongoing [90]. Studies suggest that teachers need to be adept at identifying, analyzing, and solving problems to address diverse student needs and classroom contingencies [91]. Teachers with strong problem-solving skills can design and implement teaching activities more effectively, promoting students’ deep learning and application abilities. In recent years, the development of educational technology has provided teachers with new tools and resources to better address teaching challenges [92,93]. For example, AI-based online teaching platforms and educational applications offer rich teaching resources and student data, helping teachers better understand student needs and adjust teaching strategies. Teachers’ problem-solving skills are not only reflected in classroom teaching but also in interactions with students, parents, and colleagues, helping to address various educational challenges and improve teaching quality and student performance.

In the AI era, characterized by information overload, the burden of distinguishing between true and false information and the significant differences in value content pose cognitive challenges for education and learning. Hence, teachers particularly need critical thinking skills to face these challenges. Critical thinking is not only a professional skill for teachers but also a habit of mind, cultivated through systematic training and practice [94]. It includes the abilities to interpret, analyze, evaluate, and reason, which not only aid in academic success but also enhance decision-making and problem-solving abilities in daily life [95]. By designing challenging teaching activities and encouraging students to engage in critical thinking, teachers can effectively promote the development of students’ critical thinking skills. Applying critical thinking in teaching helps students question existing knowledge systems, fostering their inquiry spirit and problem-solving abilities.

Creative thinking is key to promoting educational innovation and student development [96]. Students’ creative thinking is closely related to their teachers’ creative thinking; a teacher lacking in creative thinking is unlikely to cultivate creative students [97]. Due to differences in theoretical basis, judgment criteria, research methods, and focus, scholars have defined innovative abilities in various ways. The “4C” model proposed by Kaufman and Beghetto [98], encompassing critical thinking and problem-solving, communication, collaboration, and creativity and innovation, has gained wide recognition. Existing research shows that teachers can significantly enhance students’ creative thinking abilities through innovative teaching activities [99,100]. By designing innovative teaching activities and encouraging students to propose novel solutions, teachers can effectively foster students’ creativity. Teachers’ creative thinking not only contributes to students’ academic success but also enhances their competitiveness in future careers. In the teaching process, teachers should focus on cultivating students’ creative thinking by providing open-ended questions and encouraging innovative thinking to promote their creativity.

Collaboration holds an important position in 21st-century teachers’ teamwork and interdisciplinary cooperation [101]. Teacher collaboration involves experience sharing, mutual learning, and resource sharing regarding practical teaching issues within educational activities and workplaces. Research defines AI collaboration as enhancing students’ subject knowledge and abilities through data-driven problem-based learning, case-based reasoning, and growth- and reflection-oriented assessments [102]. Teamwork not only improves teachers’ job satisfaction and collective strength but also promotes the overall improvement of teachers’ qualities and abilities [103]. Individual professional development relies on group communication, cooperation, mutual trust, and assistance, while collective growth inevitably drives more teachers’ professional development. Long-term activities such as collective lesson preparation, team lesson polishing, group problem-solving, and team research projects have improved the entire teaching staff’s professional quality and level.

**Figure 1:**
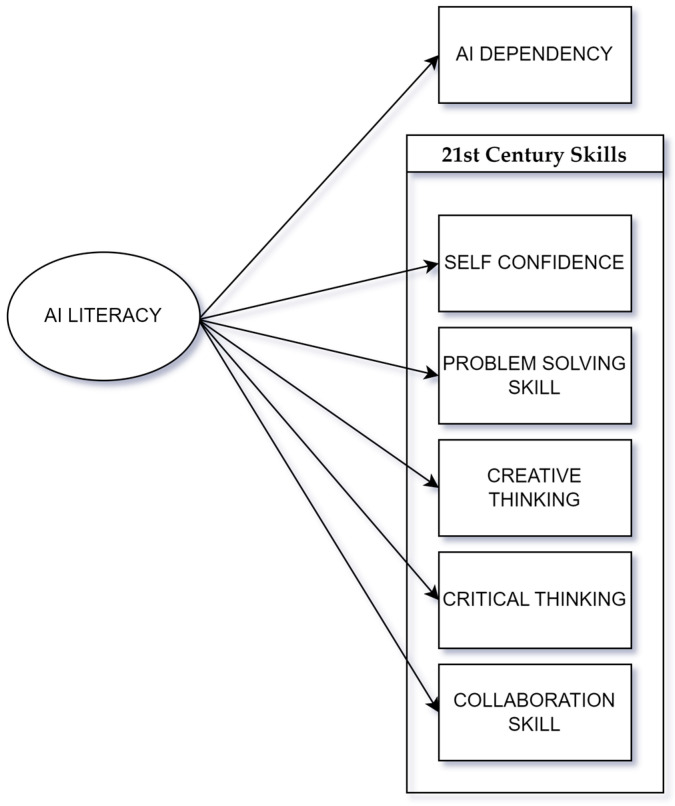
Conceptual model of the relationships between AI literacy, AI dependency, and 21st-century-skill development.

Empirical evidence and theoretical frameworks, as detailed in Figure 1, show that AI technology can significantly enhance crucial 21st-century skills in education. This study introduces a theoretical framework to explain the relationships between AI literacy, trust, AI dependency, and 21st-century skills. Hypothesis 1 proposes that higher AI literacy among mathematics teachers correlates with greater dependency on AI technologies. As teachers become more familiar and competent with AI tools, they are more likely to trust and rely on these technologies in their teaching practices, increasing their overall dependency. Hypothesis 2 posits that AI literacy positively influences the development of 21st-century skills, such as critical thinking, problem-solving, and collaboration. Teachers who are more literate in AI are better equipped to use these tools to foster skill development in students. However, excessive AI dependency could reduce these advantages, potentially limiting opportunities for critical thinking and problem-solving. Therefore, a balanced approach to AI integration is essential to ensure that AI supports, rather than replaces, the core teaching skills necessary for students’ future success.

## 3. Methodology

In this section, the methodology adopted to achieve the objectives of this study is explained. First, the instruments used in the research are described. Second, in the data collection section, the steps taken to gather the data are detailed. Third, in the data analysis section, the procedures for processing the data to meet the research objectives are outlined.

### 3.1. Instruments

#### 3.1.1. General Information

In the initial section of the instrument, essential information about the mathematics teachers was gathered. This included sociodemographic details such as gender, age, educational level, length of AI use, and place of residence.

#### 3.1.2. AI Literacy

The AI literacy instrument was adapted from the research presented in [104,105,106] and was customized to align with the specific characteristics of mathematics teachers in China. It assesses individual AI literacy levels using a 5-point Likert scale that ranges from ‘strongly disagree’ to ‘strongly agree’. The instrument’s reliability was confirmed by achieving a Cronbach’s alpha of 0.91.

#### 3.1.3. AI Trust

The AI trust questionnaire was adapted from the research presented in [107,108]. It is divided into two variables: accepting AI and fearing AI. The questionnaire utilizes a 5-point Likert scale, with some items scored in reverse. In this study, the AI trust questionnaire achieved a Cronbach’s alpha of 0.86.

#### 3.1.4. AI Dependency

The AI dependency questionnaire was developed from descriptions of compulsive behaviors or dependencies in the DSM-5 presented in [13,14]. It includes three criteria for AI dependency: Feeling of Vulnerability, Concern about Relevance and Performance, and Seeking External Validation, indicating an emotional dependence on AI tools for decision-making. The AI dependency questionnaire also uses a 5-point Likert scale. The reliability of AI dependency was shown by an excellent Cronbach’s alpha of 0.95.

#### 3.1.5. Twenty-First-Century Skills

According to UNESCO, 21st-century skills encompass eight objectives [109]. These skills include critical thinking and problem-solving, which empower individuals to tackle complex issues with strategic solutions. Collaboration and teamwork are emphasized for their importance in working effectively within diverse groups. Creativity and innovation are highlighted as necessary for generating new ideas and approaches. Global citizenship involves an understanding of global issues and cultural diversity. Digital literacy is crucial for effectively utilizing technology to manage and create information. Effective communication skills are necessary for clearly expressing ideas across various mediums. Lifelong learning and self-direction are essential for ongoing personal and professional development. Lastly, environmental literacy equips individuals to make informed decisions that consider ecological impacts. This study adopted the five most relevant 21st-century skills for mathematics education and teachers: self-confidence, problem-solving, critical thinking, creative thinking, and collaboration skills. The questionnaires for these five variables were adapted from previous research [110,111,112,113]. All of them utilize a 5-point Likert scale. The Cronbach’s alpha for the five variables was 0.89. (The reported Cronbach’s alpha of 0.89 represents the overall internal consistency for all items combined across these sub-skills, not separate alpha values for each individual sub-skill. This approach was chosen to ensure a comprehensive assessment of the interrelated constructs as they pertain collectively to the context of mathematics education).

### 3.2. Data Collection and Participants

In this study, a purposive sampling strategy was employed to select mathematics teachers from Qinghai Province, China, a province known for its commitment to integrating AI tools into educational practices. The sample included teachers from both primary and secondary schools across the province who had participated in seminars and workshops in July 2023 on AI tools in mathematics education organized collaboratively by the Qinghai government and Qinghai Normal University. This ensured that the teachers in the study were not only exposed to but also actively implementing AI technology in their educational routines, such as lesson planning and task execution.

Eligibility for inclusion required teachers to have completed the AI technology course in July 2023 and to have been applying this knowledge in their teaching for at least one year. This criterion was set to ensure that participants had a sufficient level of familiarity and proficiency with AI tools, reflective of a group that embodies progressive educational practices in the region.

The selection of schools and teachers was guided by accessibility and the willingness to participate, which are crucial in a province characterized by significant geographical and economic diversity. This approach accommodated the practical constraints of conducting field research in Qinghai while ensuring a relevant and informed participant base.

Considering the busy schedules of mathematics teachers and to ensure the anonymity of the questionnaire, it was distributed via the online platform Wenjuanxing. Data collection was conducted over a three-week period in May 2024. After an initial screening, 19 low-quality data points (e.g., straight-line responses) were removed from the study. From the remaining 469 respondents, 68% (319) were female and 32% (150) were male. Of these, 59% (277) lived in urban areas while 41% (192) resided in rural areas. The majority (91%, 425 respondents) held undergraduate degrees, and 9% (44) had postgraduate qualifications. The average age of the participants was 23.87 years (SD = 1.34).

### 3.3. Research Ethics

Ethical approval was obtained from School of Mathematical Sciences, Beijing Normal University, Beijing, China (MS202404038, 3 April 2024). prior to the commencement of data collection. At the beginning of the questionnaire, detailed explanations about the purpose of this study were provided. Also, it was clarified that the data collected would only be used for research purposes and would be kept confidential. Participants were invited to voluntarily participate in the study. It was emphasized that there were no risks associated with participation and that participants were free to withdraw from the study at any time without any consequences.

In line with ethical guidelines such as the Helsinki Declaration, these measures ensure that all participants are fully informed of the nature of the study and consent to participate under conditions that respect their privacy and autonomy. This approach not only adheres to ethical standards but also fosters trust between the researchers and participants, ensuring that the data collected are both reliable and ethically obtained.

### 3.4. Data Analysis

Data analysis was conducted using a three-step procedure [114]. First, descriptive statistics were calculated, and correlations among the variables under investigation were tested. Next, confirmatory factor analysis (CFA) was performed to assess the construct validity of the variables used in this study. The second step involved using latent profile analysis (LPA) to identify different profiles based on the AI literacy and AI trust of mathematics teachers. The characteristics of each profile were then analyzed. The final step was to compare the differences in AI dependency and 21st-century skills among the profiles.

Mplus 8 was used to conduct the LPA analysis. Mplus 8, a statistical analysis software widely recognized for its capabilities in handling complex models, was used to conduct the LPA in this study. Developed by Muthén and Muthén, Mplus offers advanced techniques in structural equation modeling, multilevel modeling, and longitudinal data analysis, making it particularly suited for dissecting the intricate relationships within educational data. The goal of the latent profile analysis (LPA) was to identify the optimal number of profiles based on AI literacy and AI trust among mathematics teachers. To determine the most suitable model, we evaluated several statistical criteria including the Akaike Information Criterion (AIC), Bayesian Information Criterion (BIC), and sample-adjusted Bayesian Information Criterion (SABIC), where lower scores suggest a better model fit [91,115]. Furthermore, a significant *p*-value in the Vuong–Lo-Mendell–Rubin Likelihood Ratio Test or LRT suggests that the k-class model fits significantly better than the k-1 model and should be selected [116]. Additionally, we assessed model clarity using entropy values, where values closer to 1 indicate a clearer classification of individuals into profiles [117]. In instances where statistical indicators suggested multiple valid models, we prioritized the model that best aligned with theoretical expectations and ensured that each identified profile included a sufficient number of individuals to maintain robustness in our analysis [118].

Finally, to fulfill the second objective of our study, Analysis of Variance (ANOVA) was employed to methodically examine the differences in AI dependency and self-confidence, problem-solving, critical thinking, creative thinking, and collaboration among the various profiles identified through LPA. The ANOVA helped to assess whether the different levels of AI literacy and trust profiles correspond to variations in AI dependency and the development of self-confidence, problem-solving, critical thinking, creative thinking, and collaboration among mathematics teachers.

Variations in AI dependency and essential skills such as self-confidence, problem-solving, critical thinking, creative thinking, and collaboration, which are vital for 21st-century educators, were specifically identified. The *p*-value was set at a threshold of <0.05.

## 4. Results

### 4.1. Preliminary Analysis

Table 1 presents descriptive statistics and bivariate correlations among variables in this study. The correlation analysis indicates that both AI literacy and trust in AI are significantly correlated with AI dependency, as well as negatively associated with self-confidence, problem-solving, critical thinking, creative thinking, and collaboration skills. Furthermore, AI dependency is closely linked to declines in self-confidence and the aforementioned skills, illustrating the complex interactions between these psychological constructs and the adoption of AI in an educational setting.

To measure the construct validity of the study’s latent variable measurements, we conducted a comprehensive confirmatory factor analysis (CFA). This analysis included all the study variables: AI literacy, AI trust, and intervention outcomes (AI dependency, self-confidence, problem-solving, critical thinking, creative thinking, and collaboration skills). The results of the CFA indicated excellent model fit with a Comparative Fit Index (CFI) of 0.837, Tucker–Lewis Index (TLI) of 0.871, Root Mean Square Error of Approximation (RMSEA) of 0.027 [0.016, 0.034], and Standardized Root Mean Square Residual (SRMR) of 0.021 [119]. All standardized factor loadings were significant, ranging from 0.625 to 0.912.

### 4.2. Latent Profile Analysis of the AI Literacy and AI Trust of Mathematics Teachers

The LPA approach was applied by running models ranging from one to six profile solutions to identify the most appropriate model for categorizing mathematics teachers based on their AI literacy and trust (refer to Table 2 for details). The results indicated that the five-profile solution yielded the highest entropy among the models. This high entropy value suggests that the classification of individuals into different profiles was particularly clear and distinct in the five-profile model, enhancing the reliability of profile assignments.

While the six-profile solution presented lower values for AIC, BIC, and SABIC, indicating a potentially better fit due to more granularity in the profiles [117], the LMR (Lo–Mendell–Rubin) *p*-value for this model was 0.20. This *p*-value is significantly higher than the typical threshold of 0.05 used for determining statistical significance in model comparisons. A higher LMR *p*-value implies that the addition of a sixth profile does not significantly improve the model fit compared to the five-profile solution. Therefore, based on the combination of entropy values and the LMR test results, it is concluded that expanding beyond five profiles does not offer additional meaningful differentiation among the groups of teachers. Thus, the five-profile solution was adopted as it balances model fit and interpretability, providing a clear and statistically supported structure for understanding the variations in AI literacy and trust among mathematics teachers.

**Figure 2:**
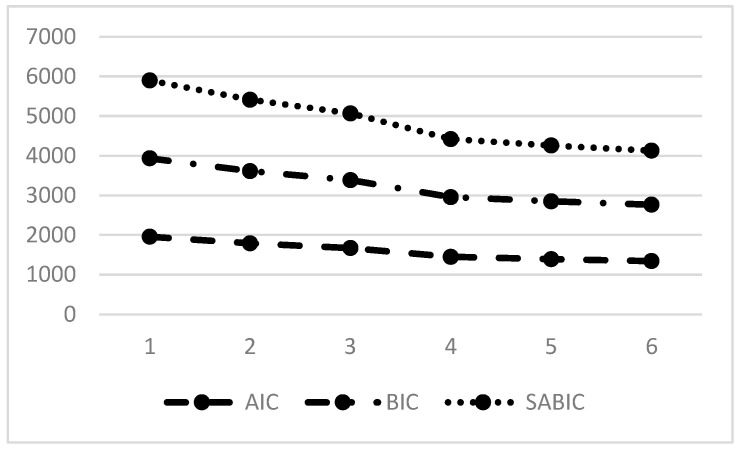
Plot of model fit indices (latent profile analysis).

Figure 2 illustrates that the AIC, BIC, and SABIC values decreased as more profiles were added, indicating improved model fit with increased complexity. However, this improvement plateaued after the four-profile model, as further decreases in these indices were minimal. The highest entropy, indicating the clearest classification of cases, occurred with the five-profile solution. Despite the slightly lower AIC, BIC, and SABIC values in the six-profile model, its higher LMR *p*-value of 0.20 suggests no significant improvement over the five-profile solution. Therefore, considering the balance of statistical fit, clarity of classification, and interpretability, the five-profile model is identified as the optimal choice for understanding variations in AI literacy and trust among mathematics teachers.

**Figure 3:**
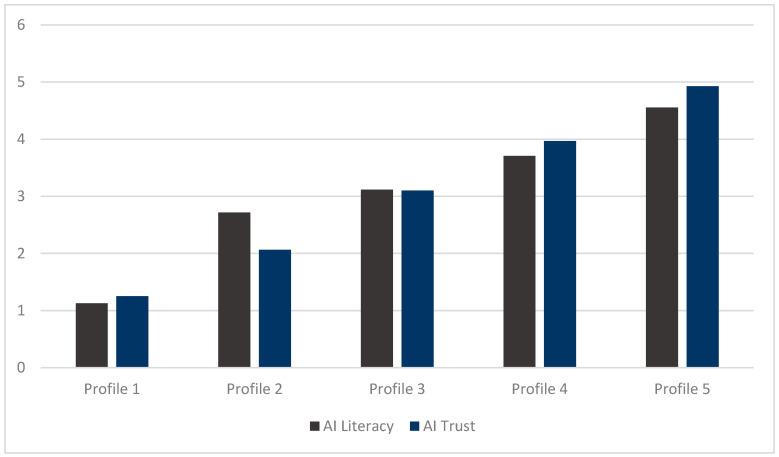
Mean of the four dimensions of AI literacy and AI trust of mathematics teachers for each profile.

Figure 3 displays the five profiles based on the average scores of AI literacy and AI trust of mathematics teachers for each profile (see Table 3 for details). Each profile is named according to the average score of AI literacy of mathematics teachers. Thus, the five profiles are named as follows: (1) Basic AI Engagement, (2) Developing AI Literacy, Skeptical of AI, (3) Balanced AI Competence, (4) Advanced AI Integration, and (5) AI Expertise and Confidence.

Profile 1, named Basic AI Engagement (BAE), features teachers with low levels of AI literacy and trust. This profile likely includes teachers who are less familiar or comfortable with AI technologies, potentially due to limited exposure or skepticism about AI’s effectiveness in education. Although there were only four such teachers in our sample (representing approximately 0.85% of the total sample of 469), this group highlights a minimal yet significant segment of the population that may require targeted interventions to boost AI engagement.

Profile 2, named Developing AI Literacy, Skeptical of AI (DAL-SAI), consists of 16 teachers (3.41% of the total) with medium AI literacy but lower trust in AI. These teachers may recognize the potential of AI tools but remain cautious due to concerns about dependability and ethical implications.

Profile 3, named Balanced AI Competence (BAC), includes 133 (28.36%) teachers with moderate levels of both AI literacy and trust. This profile represents a well-balanced approach to AI, where teachers are sufficiently skilled and confident to integrate AI tools effectively into their teaching practices.

Profile 4, named Advanced AI Integration (AAI), is characterized by 269 teachers (57.36%) with medium–high levels of both AI literacy and trust. These educators are likely to be early adopters who actively integrate AI into their pedagogy and may serve as role models or mentors in promoting AI within their institutions.

Profile 5, named AI Expertise and Confidence (AIEC), includes 47 teachers who exhibit high levels of both AI literacy and trust. These teachers are not only proficient in using AI tools but also highly trust their functionality and impact, likely driving innovation and best practices in AI integration. At 10.02% of the total sample, this profile indicates a group of leading-edge educators who could spearhead professional development and advocacy efforts in AI education.

### 4.3. Results of AI Dependency, Self-Confidence, Problem-Solving, Critical Thinking, Creative Thinking, and Collaboration in Each Latent Profile

**Figure 4:**
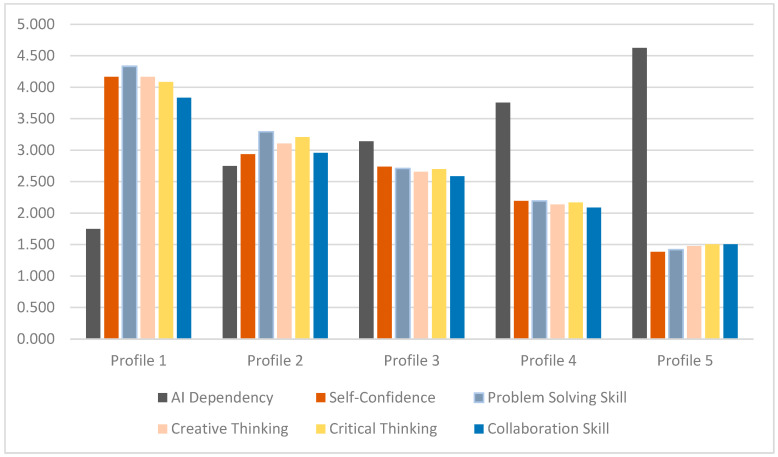
AI literacy and trust and their relationship with AI dependency and 21st-century skills.

To achieve the second objective of the study, which is to determine if differences in AI literacy and AI trust levels correspond to differences in AI dependency and 21st-century skills, a series of ANOVAs were conducted. Table 4 and Figure 4 display the descriptive statistics of the means across the five profiles, as well as the results from the ANOVAs and post hoc comparisons.

As illustrated in Table 4, the ANOVA results reveal significant differences across the five profiles regarding AI dependency and essential 21st-century skills including self-confidence, problem-solving, critical thinking, creative thinking, and collaboration. The data exhibit a concerning trend: while AI dependency increases from Profile 1 (Basic AI Engagement) to Profile 5 (AI Expertise and Confidence), the levels of vital 21st-century skills correspondingly decrease.

This inverse relationship is statistically significant, suggesting that higher AI literacy and trust lead to greater AI dependency but adversely affect the development of critical educational skills. For example, the ANOVA for AI dependency shows significant effects (F [3, 357] = 91.8, η^2^ = 0.442, *p* < 0.001), indicating that as profiles increase in AI dependency, the proficiency in skills such as critical thinking declines significantly (F [3, 357] = 84.941, η^2^ = 0.423, *p* < 0.001). Similarly, problem-solving skills decrease as the profiles ascend, with Profile 5 scoring lowest despite high AI literacy (F [3, 357] = 108.346, η^2^ = 0.483, *p* < 0.001).

These results underscore a pivotal challenge: although AI can enhance certain aspects of educational engagement, an over-reliance on these technologies may hinder the development of crucial human-centric skills. Therefore, it is essential to strike a balance in AI integration, ensuring that while educators leverage technology for efficiency and engagement, they also maintain and develop the interpersonal and cognitive skills that are critical for 21st-century education.

The results indicate that profile membership explains approximately 44.2% of the variance in AI dependency, 42.9% in self-confidence, 48.3% in problem-solving, 42.3% in critical thinking, 41.6% in creative thinking, and 35.1% in collaboration skills. These percentages, denoted by the eta-squared (η^2^) values, reflect the significant influence that different levels of AI literacy and trust within each profile have on these key educational and professional capabilities.

## 5. Discussion

This research employed the latent profile analysis (LPA) approach to identify distinct profiles of mathematics teachers based on their AI literacy and trust. Further analysis explored differences in AI dependency, self-confidence, problem-solving, critical thinking, creative thinking, and collaboration across these profiles. Unlike previous studies that focused on variables, this study emphasizes individuals, analyzing their responses to various questions and examining the dimensions of AI dependency and 21st-century skills. Our findings reveal five profiles of AI literacy and trust of mathematics teachers. As AI literacy and trust increased, so did AI dependency among mathematics teachers, accompanied by a significant decline in AI dependency, self-confidence, problem-solving, critical thinking, creative thinking, and collaboration.

The five latent profiles of AI literacy and trust of mathematics teachers and their characteristics are detailed as follows:

Our research supports the premise that mathematics teachers possess multiple dimensions of AI literacy and trust simultaneously. Additionally, our study is the first to identify profiles of AI literacy and trust of mathematics teachers, while previous research which focused more on the effects of AI technology often centered on how AI applications influence teaching practices and student outcomes [15,120], neglecting the varied personal capabilities and attitudes of the teachers themselves towards AI. These studies generally looked at direct impacts such as efficiency in grading, personalized learning opportunities for students, and enhanced instructional methods. Based on the sample in this study, a five-profile solution that best fits the data was identified. This finding contrasts with previous LPA studies, which often identified no more than three profiles [121,122]. Based on the findings of this study, a possible portrait of the characteristics of teachers of the five profiles might be as follows:

AI Expertise and Confidence (AIEC) characterizes mathematics teachers who demonstrate high levels of AI literacy and trust in technology. Typically, teachers in this profile are experienced male mathematics educators who have integrated AI technology into their teaching. Their strong belief in the positive impacts of AI on student engagement and success in mathematics is evident. However, despite their high AI literacy and trust, the results of this study indicate that teachers with the AIEC profile exhibit the highest levels of AI dependency. This high reliance on AI technology needs to be carefully managed. Overdependence on AI technology is a double-edged sword [56,123]; while it assists in mathematics learning activities and can dramatically improve instructional quality, it could also lead to negative effects in long-term use. For instance, excessive reliance on AI might diminish teachers’ own problem-solving skills and reduce direct student–teacher interactions, potentially impacting critical thinking development in students. Researchers hypothesized that over time, the pervasive use of AI technology could fundamentally alter the teaching dynamics and the nature of the learning activities in mathematics. This shift might result in a transformation of the educational landscape, where digital proficiency becomes as crucial as mathematical skills, possibly reshaping educational priorities and outcomes.

The Advanced AI Integration (AAI) profile comprises mathematics teachers who exhibit medium to high levels of both AI literacy and trust. Teachers in this profile have slightly lower AI literacy compared to those in the AIEC profile. They also demonstrate somewhat lower levels of trust in AI technologies than their AIEC counterparts. Basic information reveals that teachers within the AAI group occasionally engage in traditional, non-AI-driven mathematics teaching activities.

Balanced AI Competence (BAC) comprises mathematics teachers with moderate levels of both AI literacy and trust. They use AI technology as a supplement and balance it with traditional teaching methods rather than relying solely on technology. These educators prioritize balancing traditional teaching methods with AI tools, using technology to supplement, rather than replace, core mathematical teachings.

The Developing AI Literacy, Skeptical of AI (DAL-SAI) profile includes mathematics teachers who possess medium levels of AI literacy but exhibit lower trust in the reliability and ethical implications of AI. Basic information about these teachers indicates that while they recognize the potential benefits of AI in mathematics education, they remain cautious about becoming overly dependent on such technology. Despite their moderate proficiency in AI, these teachers continue to express concerns about the potential negative impacts of AI usage in educational settings.

Basic AI Engagement (BAE) features mathematics teachers with low levels of AI literacy and trust, which may stem from a lack of exposure to effective AI tools or skepticism about their relevance to mathematics education. However, their low AI literacy and trust result in minimal dependency on AI technology. Notably, teachers in this profile exhibit the highest levels of self-confidence, problem-solving, critical thinking, creative thinking, and collaboration skills compared to the other four mathematics teacher profiles. While the instructional practices of teachers within the BAE profile may be less integrated with AI technology, they demonstrate a significant advantage in essential educational competencies. Their low dependency on AI fosters a strong foundation in traditional educational skills and interpersonal attributes, which are critical for effective teaching and learning environments. This highlights the importance of balancing technological tools with the development of foundational teaching skills to ensure a holistic educational approach.

It should be noted that while other studies have found that AI technology positively affects teacher abilities and advocate for increasing AI literacy [2,58], this research demonstrates that higher AI literacy and trust do not always lead to beneficial impacts for mathematics teachers. An ideal profile seems to be one with appropriate levels of AI literacy and trust, where mathematics teachers may balance the use of AI technology in the classroom without becoming overly dependent on it. By understanding the diverse levels of AI literacy and trust among mathematics teachers, educational leaders and policymakers can tailor professional development programs that address the specific needs and reservations of teachers in each profile. This targeted approach ensures that mathematics teachers are equipped to integrate AI into their teaching practices effectively, enhancing educational outcomes while preserving the integrity of mathematics education.

The findings emphasize the need for a nuanced approach to integrating AI in educational settings, considering that the benefits of AI are maximized only when teachers maintain a critical and balanced perspective towards its use. Educational systems should focus on developing programs that not only improve AI literacy but also educate teachers about the ethical dimensions and limitations of AI. Professional development should encourage teachers to explore diverse teaching tools, ensuring that AI is one of the many strategies that could be employed to enhance student learning. Additionally, continuous feedback from teachers about their experiences with AI can guide the ongoing adaptation of educational technologies to better suit classroom realities, fostering an educational landscape that values both human insight and technological advancement.

**Increasing AI Literacy and Trust Significantly Correlates with Higher AI Dependency**

Our findings indicate a significant correlation between increased levels of AI literacy and trust among mathematics teachers and their dependency on AI technologies. This research complements previous studies on the negative effects and concerns to be aware of as AI technology develops and becomes more widely used in education [124,125]. As teachers become more literate and trusting of AI, they tend to rely more heavily on these tools in their teaching practices. This relationship suggests that as mathematics teachers become more comfortable with AI capabilities, they increasingly integrate these technologies into their curriculum, potentially relying on AI for tasks such as data analysis, automated grading, and personalized learning experiences. However, this integration must be approached with caution to avoid an excessive dependency that could diminish pedagogical richness and reduce personal engagement with students.

However, while this integration can streamline certain aspects of teaching and potentially enhance learning outcomes, it also raises important considerations about the balance of technology use in education [82,126]. High dependency might lead to an over-reliance on AI [14], where mathematics teachers might defer too much to technology for instructional decisions, potentially at the expense of pedagogical richness and personal engagement with students. This underscores the need for ongoing professional development programs to be structured not only to enhance AI literacy and trust but also to educate teachers on critical engagement with AI. These programs should focus on developing skills for discerning when and how AI tools can be most effectively integrated into teaching, ensuring that technology serves as an aid rather than a crutch. Practical sessions could include scenario-based training that simulates decision-making processes with and without AI assistance, helping teachers to visualize the benefits and limitations of AI in real-time teaching contexts.

**Increasing AI Literacy and Trust Significantly Correlates with the Decline of 21st-Century Skills**

Our study finds that as mathematics teachers become more familiar and confident with AI tools (AI literacy and trust), they tend to use these tools more often, which we refer to as AI dependency. This increased reliance on AI can lead to a decrease in important skills such as self-confidence, problem-solving, critical thinking, creative thinking, and collaboration skills. This phenomenon, termed ‘AI dependency’, suggests that while AI can facilitate certain aspects of teaching, it may also inadvertently weaken essential professional capacities.

When mathematics teachers use AI tools to complete tasks and solve mathematics problems, they may feel less self-assured in their ability to handle challenges independently, which could erode their self-confidence over time. This finding contrasts with previous research [127], which suggested that user self-confidence can influence control over AI technology usage.

Previous research has found that AI technology can help solve complex problems by providing immediate answers [128]. However, this might lead mathematics teachers to engage less in the iterative, analytical process that is essential for robust problem-solving, thus gradually impairing this skill.

Critical thinking skill is an essential ability for a teacher. It influences problem-solving, instructional strategies, and teachers’ professional development. Previous findings suggest that respondents believed that AI could enhance critical thinking skills [129]. However, this differs from the findings in our study. We found that AI may limit teachers’ exposure to complex problem contexts where critical thinking is required, thereby reducing opportunities to develop and refine this skill.

Some literature indicates that AI technology can negatively affect creative thinking skills [130]. This study aligns with this previous research, suggesting that the prescriptive nature of many AI applications may discourage creative explorations and lead to a decline in the ability to generate novel solutions and ideas.

Some AI tools support individual and rapid task completion, reducing the need for collaboration [131]. This shift towards solitary work can have profound implications in educational settings where collaboration is often essential. When teachers rely on AI for instructional and administrative tasks, the decreased necessity for collaborative planning can lead teachers to work more in isolation. Over time, this can weaken their ability to effectively collaborate with colleagues, which is critical for developing and sharing innovative teaching strategies and refining pedagogical approaches, ensuring a comprehensive educational experience for students.

The discussion above highlights a critical need for educational strategies that not only encourage the development of AI competencies among teachers but also emphasize the importance of integrating AI in ways that support and not supplant the development of 21st-century skills. Educators should be guided to use AI as an aid rather than a replacement for their instructional skills [132], policymakers should consider guidelines that encourage judicious use of AI in educational settings. These guidelines could recommend regular reviews of AI usage in classrooms, ensuring that it is used as a complementary tool rather than a substitute for teacher engagement. By promoting a balanced approach to AI integration, we can help teachers harness technology to enhance educational outcomes while preserving and strengthening their core teaching competencies.

Additionally, professional development programs aimed at enhancing AI literacy should not only focus on how to use AI tools but also on how to critically engage with them to maintain and enhance pedagogical skills. Workshops could be designed to simulate scenarios where teachers practice switching between AI-assisted and traditional teaching methods, thereby fostering flexibility and resilience in their teaching strategies.

## 6. Limitations

This study has several limitations that warrant consideration. Firstly, the sample consists exclusively of mathematics teachers from Qinghai Province, China, which may limit the generalizability of our findings to other regions or educational contexts. The unique demographic and cultural characteristics of this sample might not reflect broader educational dynamics, particularly in places with different age distributions and cultural backgrounds. Such variations could influence not only the development of AI literacy and trust among teachers but also their perception and implementation of 21st-century skills in their pedagogy. Additionally, the conceptual frameworks of AI literacy and trust of mathematics teachers developed for this study might not be directly transferable to other educational settings without adaptation. Another concern is the use of self-reported measures, which can introduce bias into the findings. While self-reports are valuable for gaining insights into teachers’ perceptions and experiences, they are subjective and may not always accurately reflect actual behaviors or abilities. This limitation is not thoroughly addressed in the study, and future research should consider incorporating more objective measures or mixed-methods approaches to validate and enrich the self-reported data.

Additionally, the use of cross-sectional data in this research means that the insights gained reflect a snapshot in time. This approach captures the current state of AI literacy and trust among mathematics teachers but does not account for potential changes over time. The dynamic nature of technology and educational practices suggests that the profiles of AI literacy and trust identified in this study might evolve as new technologies emerge and as teachers’ experiences with AI deepen; hence, it is important to conduct follow-up studies to understand the phenomenon better over time.

## 7. Conclusions

AI technology definitely has a positive effect on mathematics learning. However, it is important to note that this study again shows that AI technology impacts AI dependency and 21st-century skills. Our research is the first to investigate the profiles of AI literacy and trust, and their relationship with AI dependency, self-confidence, problem-solving, critical thinking, creative thinking, and collaboration skills among mathematics teachers in China. Five profiles of mathematics teacher AI literacy and trust were identified. Furthermore, increases in AI literacy and trust were shown to be related to the increase in AI dependency among mathematics teachers and a decrease in AI dependency, self-confidence, problem-solving, critical thinking, creative thinking, and collaboration skills of mathematics teachers.

These findings suggest a double-edged sword of AI integration in educational settings: while AI can enhance educational practices, it can also inadvertently foster dependency, reducing the cultivation of critical 21st-century skills. To mitigate this, it is imperative that future research and practice focus not merely on the integration of AI tools but on the development of comprehensive educational strategies that leverage AI to enhance teaching effectiveness without compromising skill development.

In practical terms, this means rethinking teacher training programs to emphasize the critical evaluation of AI tools and their pedagogical implications. Curricula should be designed not only to improve AI literacy but also to integrate AI use with traditional teaching methods that promote critical and creative thinking. Additionally, policymakers should consider these dynamics as they develop guidelines and standards that govern AI use in educational settings. Future studies should explore strategic frameworks that balance AI use with the enhancement of independent problem-solving and innovative thinking skills, ensuring that AI serves as a complement rather than a substitute for robust educational practices.

## Figures and Tables

**Table 1:** Descriptive statistics and bivariate correlations.

	Variables	1	2	3	4	5	6	7	8
1	AI Literacy	1							
2	AI Trust	0.733 **	1						
3	AI Dependency	0.715 **	0.680 **	1					
4	Self-Confidence	−0.690 **	−0.665 **	−0.702 **	1				
5	Problem-Solving	−0.727 **	−0.720 **	−0.687 **	0.786 **	1			
6	Critical Thinking	−0.659 **	−0.661 **	−0.620 **	0.718 **	0.767 **	1		
7	Creative Thinking	−0.677 **	−0.666 **	−0.669 **	0.748 **	0.798 **	0.814 **	1	
8	Collaboration Skill	−0.569 **	−0.616 **	−0.552 **	0.648 **	0.647 **	0.711 **	0.715 **	1
	M	3.56	3.72	3.61	2.31	2.32	2.26	2.30	2.21
	SD	0.68	0.68	0.72	0.66	0.64	0.63	0.64	0.63

Notes: ** *p* < 0.01.

**Table 2:** Comparison of different models after latent profile analysis.

Profile No.	AIC	BIC	SABIC	LMR *p*-Value	BLRT *p*-Value	Entropy	Class Probability
1	1958.959	1975.561	1962.866	-	-	-	469
2	1791.542	1820.596	1798.379	0.00	0.00	0.715	141/328
3	1671.496	1713.003	1681.265	0.06	0.00	0.850	124/33/312
4	1450.724	1504.682	1463.423	0.03	0.00	0.958	18/135/270/46
5	1391.612	1458.022	1407.241	0.01	0.00	0.976	4/16/133/269/47
6	1343.951	1422.812	1362.510	0.20	0.00	0.952	16/4/215/100/86/48

**Table 3:** Estimated means and standard deviations for the 5-profile model (N = 469).

Profiles	Profile 1 (BAE)	Profile 2 (DAL-SAI)	Profile 3 (BAC)	Profile 4 (AAI)	Profile 5 (AIEC)
AI Literacy	1.12 ± 0.12	2.71 ± 0.14	3.11 ± 0.04	3.70 ± 0.02	4.55 ± 0.08
AI Trust	1.25 ± 0.15	2.06 ± 0.06	3.10 ± 0.01	3.96 ± 0.01	4.92 ± 0.02

**Table 4:** AI literacy and trust profiles and AI dependency and 21st-century skills, with individual ANOVA results.

	Profile 1 (BAE)	Profile 2 (DAL-SAI)	Profile 3 (BAC)	Profile 4 (AAI)	Profile 5 (AIEC)	Post Hoc Comparison	F	η^2^
AI dependency	1.75 ± 0.95	2.75 ± 0.68	3.14 ± 0.51	3.75 ± 0.54	4.61 ± 0.72	5 > 4 > 3 > 2 > 1	91.8 ***	0.442
self-confidence	4.16 ± 1.10	2.93 ± 0.78	2.73 ± 0.51	2.19 ± 0.45	1.38 ± 0.53	5 > 4 > 3 > 2 > 1	87.23 ***	0.429
problem-solving	4.33 ± 0.81	3.29 ± 0.60	2.70 ± 0.46	2.19 ± 0.44	1.41 ± 0.49	5 > 4 > 3 > 2 > 1	108.346 ***	0.483
critical thinking	4.16 ± 1.10	3.10 ± 0.86	2.65 ± 0.48	2.13 ± 0.42	1.47 ± 0.54	5 > 4 > 3 > 2 > 1	84.941 ***	0.423
creative thinking	4.08 ± 1.06	3.20 ± 0.67	2.69 ± 0.51	2.16 ± 0.44	1.50 ± 0.58	5 > 4 > 3 > 2 > 1	82.589 ***	0.416
collaboration	3.83 ± 1.37	2.95 ± 0.80	2.58 ± 0.58	2.08 ± 0.40	1.50 ± 0.62	5 > 4 > 3 > 2 > 1	62.611 ***	0.351

Note: *** indicates <0.01.

## Data Availability

The data are currently not publicly available due to participants’ privacy, but they are available from the first author upon reasonable request.
